# A Description of Laying Hen Husbandry and Management Practices in Canada

**DOI:** 10.3390/ani8070114

**Published:** 2018-07-11

**Authors:** Nienke van Staaveren, Caitlin Decina, Christine F. Baes, Tina M. Widowski, Olaf Berke, Alexandra Harlander-Matauschek

**Affiliations:** 1 Department of Animal Biosciences, Ontario Agricultural College, University of Guelph, Guelph, ON N1G 2W1, Canada; nvanstaa@uoguelph.ca (N.v.S.); cbaes@uoguelph.ca (C.F.B.); twidowsk@uoguelph.ca (T.M.W.); 2 Department of Population Medicine, Ontario Veterinary College, University of Guelph, Guelph, ON N1G 2W1, Canada; cdecina@uoguelph.ca (C.D.); oberke@uoguelph.ca (O.B.)

**Keywords:** aviary, furnished cage, floor system, management, housing, poultry

## Abstract

**Simple Summary:**

Furnished cage and non-cage (single-tier or multi-tier) housing systems are increasingly used worldwide in efforts to improve laying hen welfare. Canadian laying hen farms are undergoing a similar transition, however, little is known about the housing and management of laying hens in these housing systems in Canada. Data collected through farmer questionnaires from 65 laying hen flocks across Canada revealed commonly used management practices in furnished cage (26), single-tier (17) and multi-tier systems (22). Non-cage systems should allow hens to perform natural behavior (e.g., foraging/dustbathing). However, a proportion of non-cage systems either did not provide litter or considered manure as a litter substrate, which could have implications for consumer perspectives on these systems. Daily flock inspections and vaccination schemes were the main practices used to maintain flock health, whereas veterinarian involvement on-farm and in the development and implementation of a flock health plan was less common. Further research is needed to make clear recommendations and to investigate how to facilitate management changes by farmers currently transitioning to furnished cage and non-cage housing systems.

**Abstract:**

Canadian laying hen farms are transitioning from conventional cage housing to furnished cage and non-cage housing systems to improve laying hen welfare. However, little is known about the current housing and management systems in Canada. This study addresses this knowledge gap by describing different housing and management practices used on farms where laying hens were housed in furnished cages or non-cage housing systems. A questionnaire covering farm and housing conditions, litter management, nutrition and feeding, environmental control, flock characteristics, rearing and placement, health, egg production and performance were distributed through provincial egg boards to 122 producers across Canada. Data were collected from 65 laying hen flocks (52.5% response rate) in 26 furnished cage, 17 single-tier and 22 multi-tier systems. Flocks were on average 45.1 ± 14.59 weeks old (range: 19–69 weeks). Frequencies of different management practices were calculated according to housing system. Most flocks were reared in the same housing system as they were housed in during lay, with the exception of furnished cage layers which were reared in conventional cage systems. Results indicated that a large proportion of non-cage systems were either fully slatted or had manure as a litter substrate, which could have implications for consumer perspectives on these systems. Further research is needed to develop clear recommendations on proper litter management for farmers. In general, flock health was managed through daily inspections and vaccination schemes, whereas veterinarian involvement on-farm was less common. Vaccination, hygiene, and effective biosecurity should be maintained to ensure good health in laying hens in furnished cage and non-cage systems during the transition to these systems.

## 1. Introduction

To improve laying hen welfare, housing systems, such as furnished cage and non-cage housing systems, are becoming increasingly popular worldwide. Conventional cages have been banned in the EU since 2012 (Council Directive 1999/74/EU) due to their restriction on birds’ natural behavior and movement [1]. Several countries have committed to ban furnished cages and will transition to complete non-cage production in single-tier or multi-tier systems [2,3]. Similar to Europe, movements in the USA, Australia, and Canada have led some of the world’s largest restaurant chains and companies to declare their intent to only use non-cage eggs due to public demand [2,4,5,6].

In Canada, the egg industry is regulated through a supply management system in which production, pricing, and import of eggs is managed in order to maintain stable prices for farmers and consumers [7]. With changes in animal welfare policies of large restaurant chains and companies, the egg industry has to anticipate future market directions and adapt accordingly. Interestingly, von Massow et al. [8] found that restaurants also play a large role in increasing consumer awareness of choices and in shaping consumer behavior in grocery stores, which could further increase consumer demand for non-cage eggs in Canada.

The Egg Farmers of Canada (EFC) have announced a “coordinated, systematic, market-oriented transition from conventional egg production toward alternative systems, i.e., furnished cages and non-cage systems (single-tier or multi-tier systems)” that is to be completed by 2036 [9]. At the time of the announcement, 90% of egg production on the more than 1000 Canadian egg farms occurred in conventional cage housing systems [9]. With over 24 million laying hens in production in Canada [10], the transition of these farms to furnished cage and non-cage housing systems is a large-scale challenge. Additionally, the recently updated Canadian Code of Practice for the Care and Handling of Pullets and Layers, which came into effect in March 2017, has set detailed interim and final requirements for space, nests, perches and litter during the transition period [11]. All Canadian farmers will have to adapt their management to these new housing systems in order to meet these requirements. However, little scientific information is available about the development of housing and management practices for laying hens in furnished cages or non-cage systems in Canada. The goal of this study was to describe different housing and management practices being used on egg production farms across Canada where laying hens were housed in furnished cages or non-cage (single-tier or multi-tier) systems as assessed in laying hen flocks placed between October 2016 and December 2017.

## 2. Materials and Methods

A questionnaire was designed as part of a larger cross-sectional study to identify housing and management factors associated with feather damage in laying hens kept in furnished cage and non-cage housing systems on Canadian farms [12]. The questionnaire was available in both French and English. It consisted of mostly (semi-)closed questions on farm and housing conditions (including outdoor access and range use), litter management, nutrition and feeding, environmental control (e.g., lighting, air quality), flock characteristics, flock rearing and placement, flock health, egg production, and flock performance. The questionnaire was adapted from Lambton et al. [13] based on the experience of the research team with the Canadian egg production sector and input from the federal and provincial egg boards in Canada. Pilot-testing of the questionnaire was conducted at the Arkell Poultry Research Station (Ontario Ministry of Agriculture, Food and Rural Affairs [OMAFRA]—University of Guelph Agreement) as well as local farms representing the three housing systems (furnished cage, single-tier, multi-tier) to further clarify phrasing and refine the questionnaire. The data collected during pilot-testing were not included in the study. This study was approved by the University of Guelph Research Ethics Board (REB17-06-010).

Commercial laying hen farmers were invited to participate in the study through their provincial egg boards across Canada in October 2017. Invitations were sent to registered egg producers who had hens in furnished cages or non-cage systems. Producers were asked to answer the questionnaire for their current flock in these systems. Housing systems were defined as per the Code of Practice for laying hens in Canada [11]. Furnished cages refer to wire mesh enclosures with perches, nest area, scratch area and more head room compared to that available in a conventional cage. Non-cage systems refer to single-tier (barn or floor system with nests, perches, and feed and water resources located on only one level) or multi-tier systems (aviary system with nest, perching, and feed and water resources located on multiple elevated tiers). Additionally, non-cage housing systems could exist with or without outdoor access. Packages containing a cover letter, the questionnaire, and return envelope were sent out to farmers by provincial egg boards. Detailed instructions on how to fill in the questionnaire were included, as well as contact information of the research team in case of questions. Additionally, farmers were provided the opportunity to fill in the questionnaire online via Qualtrics (Qualtrics, Provo, UT, USA). All package documents were given a unique code to ensure all results were collected together. Provincial egg boards sent out a first reminder 2–4 weeks following initial distribution of the questionnaires and again two weeks before the end of data collection in December 2017.

Data were entered into Microsoft Office Excel (Professional Plus 2016) using manual double entry and were checked for entry errors. Invalid responses were considered missing values. Flocks were placed in the housing systems between October 2016 and December 2017. Space allowances were calculated based on provided data on cage dimensions and number of birds per cage. For non-cage systems, farmers were asked to provide the space allowance per bird. However, if this was not provided by farmers, average space allowance was calculated based on the dimensions of barn/housing section and the number of birds housed within this space. It should be noted that in cases where these calculations were made, it was not possible to include useable vertical space in the multi-tier systems. Frequency of the different management practices used in the different housing systems were calculated using descriptive statistics in SAS v9.3 (SAS Inst. Inc., Cary, NC, USA). Results are presented as the number and percentage of flocks for categorical variables and mean ± SD (range: min–max) for continuous variables. The number of flocks for certain variables does not necessarily add to the total number of flocks sampled in each housing system depending on farmer responses to previous questions or non-responses.

## 3. Results

A total of 122 questionnaires were sent to laying hen farmers with furnished or non-cage housing systems. Sixty-four questionnaires were returned (52.5% response rate) providing information on 65 laying hen flocks. Nearly 85% of questionnaires were returned by post, and the remaining 15% were returned through the online Qualtrics system. The majority of respondents were male (82.8%), and 50% of respondents were below 45 years of age. In the following sections, we describe the housing design and management, followed by flock characteristics, reported by respondents representing data of the 65 laying hen flocks in Canada as collected during October–December 2017. Flocks surveyed during this time period had been placed on-farm between October 2016 and December 2017, and thus, it should be noted that this spans a period where the egg production sector underwent many changes with a new Code of Practice coming into effect in March 2017 [11].

### 3.1. Housing

#### 3.1.1. Housing Design and Outdoor Access

The majority of farmers had more than 10 years of experience in egg production (52.3%) and represented all major egg production provinces in Canada with the majority located in Alberta (26.2%), British Columbia (18.5%), Manitoba (16.9%), Ontario (15.4%), and Quebec (12.3%). Twenty-six flocks were housed in furnished cages (40%), while 17 flocks (26.2%) were kept in single-tier floor systems and 22 flocks (33.8%) kept in multi-tier aviary systems. The majority of furnished cage farms had a farm size of 10,000–15,000 hens, while farms with non-cage systems had more than 25,000 hens (Table 1). Multi-tier systems consisted of two (36.4%) or three tiers (50.0%), while 13.7% had more than three tiers. Seven flocks came from organic certified multi-tier farms.

Outdoor access was provided for one flock in the single-tier and seven flocks in the multi-tier systems (20.5% of the non-cage flocks). On average, birds were 27.9 ± 2.53 weeks old when first given access to the outdoor area (range: 24.0–30.0 weeks of age). Large variation was reported in the proportion of the range that was used (61.3 ± 33.46%; range: 25.0–100.0%) and the proportion of birds that used the range (28.3 ± 17.1%; range: 1.0–50.0%). The majority of the farmers that provided outdoor access indicated that they provided resources in the outdoor area such as artificial or natural shelters (75.0%).

#### 3.1.2. Group Size and Space Allowance

**Figure 1:**
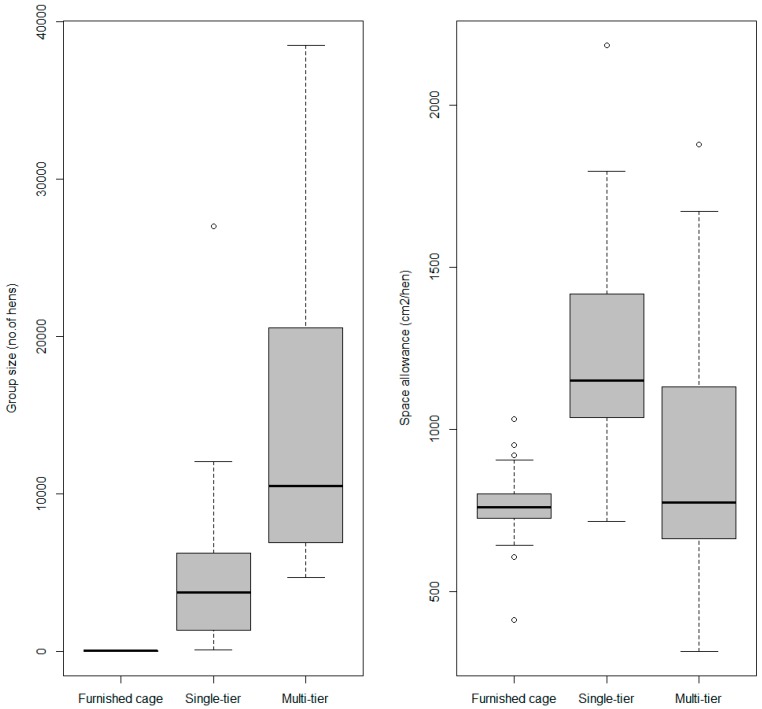
Boxplot of group size (number of hens) and space allowance (cm^2^/hen) for 65 laying hen flocks placed between October 2016 and December 2017 according to housing system (furnished cage n = 26, single-tier n = 17, multi-tier n = 22) in Canada.

Birds in furnished cages were kept in groups of 42 ± 23 hens, while birds in single- and multi-tier systems were housed in groups of 5524 ± 6858 hens and 14,732 ± 10,555 hens, respectively (Figure 1). Space allowance in furnished cages was on average 764.8 ± 121.33 cm^2^/hen. For flocks in single-tier systems, average space allowance was 1285.9 ± 396.29 cm^2^/hen, and in multi-tier systems, it was 925.4 ± 402.38 cm^2^/hen (Figure 1).

#### 3.1.3. Provision of Furnishings and Litter

Perches were provided in the majority of flocks in each housing system (Table 2). In non-cage housing systems, perches were provided at different heights, while this option was less frequent in the furnished cage housing systems. A total of 81.8% of farmers that provided perches indicated that all birds were able to perch at the same time, however it should be noted that actual perch space was not recorded in this study. Enrichment was only provided in non-cage housing systems (Table 2). Enrichment was most frequently provided in the form of hanging objects (e.g., bottles, ropes/string) (7.9%), bales of hay/straw (18.4%) or mineral blocks/pecking stones (15.8%). Birds received access to the enrichment at an average age of 24.8 ± 7.69 weeks (range: 17–42 weeks). Enrichment was provided to the majority of flocks (66.7%, 8/12 flocks) as part of standard farming practices rather than in response to specific events (e.g., cannibalism, high mortality).

Opportunities to forage and/or dustbathe are provided in the form of scratch areas in furnished cages and litter in non-cage systems. Scratch areas were provided in 53.8% of the flocks in furnished cages and were typically provided as plastic, textured mats. When flocks had a scratch area in the cage, substrate was provided (every other day to >1× per day) on the scratch area in 57.1% of the flocks. In the majority of flocks, the scratch area was cleaned ≤1× per production cycle (92.9%).

All multi-tier systems provided litter substrate, while half of the single-tier systems consisted of all wire/slatted flooring (Table 3). The proportion of the barn that was covered in litter was ≥1/3 for nearly all barns with a combination of slats, wire and litter (84.2%). The most commonly used litter substrates were wood shavings (42.9%) and sawdust (21.4%). However, there were also barns where manure was considered as litter substrate (25%). Litter was accessible upon arrival in the laying facilities in most flocks. Litter was kept at a depth of 6.7 ± 4.30 cm (range: 0.5–12.7 cm) in single-tier and 4.1 ± 2.93 cm (range: 1.3–10.2 cm) in multi-tier systems. In approximately two-thirds of the flocks, litter had not been replaced since first access (67.0%) nor raked to break up the litter (66.7%). However, this practice was more common in multi-tier systems (Table 3).

#### 3.1.4. Nutrition and Feeding

All flocks used nipple drinkers (100%), and birds were mostly fed through chain (76.2%) or pan feeders (12.7%). The majority of feed was provided in mashed form and was purchased from external sources rather than home-milled (Table 4). The number of times the diet was changed was higher in older flocks, with 0–2 changes typically occurring before 40 weeks of age, and >3 changes by the time birds reached approximately 48 weeks. In most cases, these changes in diet happened gradually (81.1%). Feeders ran on average 5.3 ± 2.24 times per day (range 2.0–12.0 times) in furnished cages, 4.8 ± 1.60 times per day (range 2.0–8.0 times) in single-tier and 6.3 ± 1.42 times per day (range 4.0–9.0 times) in multi-tier systems. The practice of midnight feeding where the dark period was interrupted to allow extra feeding (7.8%) or providing animal by-products in the diet (18.2%) only occurred in a small proportion of flocks. Supplements were provided in nearly three-quarters of the flocks (72.1%), with 18.0% providing insoluble grit, 31.2% providing insoluble fiber, 32.8% providing oyster shell, and 57.4% providing vitamins.

#### 3.1.5. Environmental Control

LED was the most common light source in laying hen flocks (Table 5), and on average, birds received 15.1 ± 0.72 h of light per day (range: 13.0–16.5 h). Light intensity as measured at bird height on the lowest level/tier, holding the light sensor horizontally facing the light source, was between 5 to 15 lux on most farms (61.2%), though it should be noted that nearly 25% of farmers did not answer this question. A dawn/dusk period was provided on most farms (83.1%), but this practice was most commonly applied on farms with furnished cages or multi-tier systems. This dawn/dusk period was typically created by the gradual dimming of lights in different areas of the barn in multi-tier systems, while in furnished cages and single-tier systems, all lights were dimmed automatically.

Average temperature and humidity were reported at 21.7 ± 1.44 °C (range: 19.0–25.0 °C) and 52.7 ± 1.74% relative humidity (RH; range: 28.0–70.0%RH), respectively. The vast majority of farmers used controlled ventilation (90.8%). Manure was removed from the barn at least once per week in furnished cage or multi-tier systems, while it was less frequently removed in single-tier systems, with the majority removing manure at the end of the flock (Table 5).

### 3.2. Flock Characteristics

#### 3.2.1. General Characteristics

On average, flocks were 45.1 ± 14.59 weeks old (range: 19–69 weeks) at the time of the study. All flocks were beak trimmed at day 1 in the hatchery using infrared beak treatment (approx. ¼ of the length of the beak was trimmed in the majority of flocks). The breed of the flocks was representative of commonly used breeds (Table 6) and were either white- (50%) or brown-feathered birds (50%). The majority of birds in furnished cages were white-feathered, while brown-feathered birds were more commonly found in non-cage housing systems.

#### 3.2.2. Flock Rearing and Placement

Most birds placed in single- or multi-tier systems were home-reared (Table 7) and consequently visited more often by laying hen farmers during rear. In multi-tier systems, all birds came from the same rearing flock, while in furnished cage and single-tier systems, some flocks were mixed. Additionally, birds were most often kept in similar housing systems during rear and lay, except for the furnished cage systems, in which birds typically came from conventional cage pullet systems. Birds were placed in the laying facilities between 14 to 20 weeks of age (mean ± SD: 18.0 ± 1.14) between October 2016 and December 2017.

#### 3.2.3. Flock Health

The majority of flocks were inspected 1–2× per day (55.4%) by 1–2 workers (95.4%) (Table 8). Approximately 70% of all farmers indicated that they always inspected the bottom and top tier and sometimes handled birds during inspection. Relatively more time was spent on daily inspections in multi-tier and furnished cage systems compared to single-tier systems. A higher amount of time spent on daily inspection was associated with a larger number of daily inspections. Approximately half of the farmers indicated that they varied the route taken during inspection. During inspections farmers assessed the health of the birds (100%), the behavior of the birds (83.1%), the functioning of equipment (96.9%), and litter quality (64.6%). Nearly all barns were disinfected before arrival of the birds (95.2%), and dedicated clothing and/or clean boot dips were used (96.8%). However, only 28.8% of farmers had developed and implemented a flock health plan with their veterinarian. No diseases had been observed since placement in the majority of flocks (82.0%). Cumulative mortality was highest in multi-tier systems with an average of 2.1 ± 0.34% (range 0.0–5.86%), compared to 1.6 ± 1.38% (range 0.0–5.23%) in single-tier systems and 1.1 ± 0.99% (range 0.02–3.68%) in furnished cages. The most frequently mentioned reasons for mortality were birds getting trapped in housing equipment (19.0%), disease (16.8%), leg injuries (15.3%), and cannibalism (13.9%).

#### 3.2.4. Egg Production and Flock Performance

Day length was increased when birds were 18.0 ± 1.33 weeks old (range: 15.5–21.0 weeks), and nearly 80% of all flocks started lay before or at 19 weeks of age. Production figures of flocks in the different housing systems are presented in Table 9. On average, birds reached 50% rate of lay at 20.9 ± 0.97 weeks of age (range: 18.0–24.0 weeks) and peak production at 26.6 ± 0.50 weeks of age (range: 22.0–36.0 weeks). Due to the range of flock ages present in the study flocks, current production rates reported at the time of the survey varied from 10.0–99.0% (mean: 89.1 ± 15.48%). Peak production rates averaged at 96.9 ± 1.76% (range: 92.0–100%) and appeared to be somewhat lower in single-tier systems compared to furnished cage systems. Average daily feed consumption per bird was higher in the non-cage systems compared to the furnished cage systems (Table 9).

## 4. Discussion

### 4.1. Housing

This study presented results of a cross-sectional survey on housing and management practices among 65 laying hen producers in Canada using furnished cages, single-tier, or multi-tier systems. This information can be used to better understand practices in these housing systems to increase farmer awareness of expected changes during the transition phase of the Canadian egg production sector. Recruitment through the provincial egg boards led to high response rates in the different provinces. It should be kept in mind that participation was voluntary, and data was collected using self-administered questionnaires, which could have biased the sample. However, the study sample included a large range of farms representing all major egg producing provinces and included a large range of farm sizes (i.e., companies and family owned farms) representative of laying hen farms in Canada. The majority of flocks were located in Alberta, British Columbia, Manitoba, Ontario, and Quebec, which are the major egg production provinces. It should be kept in mind that there are large differences in terms of climate across Canada [14], which could have implications for management practices such as ventilation, litter management, and outdoor access.

At the time of the study, approximately 28% of farms in Canada had furnished cage (11%) and non-cage housing systems (17%) present on their farm (possibly in conjunction with conventional cage systems) [15]. Our study sample therefore represented approximately 22% of those furnished cage and non-cage farms. Specifically, we received responses from around 22% of all furnished cage and 21% of all non-cage housing systems present in Canada at the time of the study [15]. Between 2016 and 2017, the number of hens housed in systems other than conventional cages increased from 16% to 23%, totaling approximately 5.6 million out of over 24 million laying hens currently in production in Canada [10,16]. In context, farmers participating in this study filled in the questionnaire for approximately 935,000 laying hens, representing nearly 17% of all hens kept in furnished cage or non-cage housing systems. The majority of respondents were male and half of the respondents were under 45 years of age, which is a somewhat higher representation of young farmers, as close to 30% of egg farmers are under 45 years of age [16]. Possibly, younger farmers were more likely to have furnished cage or non-cage systems and were more likely to participate in the study explaining this discrepancy. Additionally, the majority of farms had been working with these systems for more than one year (77.8%), while 22.2% had had their system for less than one year. This shows the current transition taking place in the Canadian egg production sector as also indicated by previous studies reporting that more than 90% of laying hens in Canada were kept in conventional cage housing a decade ago [17]. It should be emphasized that this survey spanned these fast-paced changes in the sector, together with the launch of the new Canadian Code of Practice for the Care and Handling of Pullets and Layers [11]. Some practices will be changing or will have changed already, as approximately half of the flocks were placed before the Code of Practice came into effect (placement of flocks occurred between October 2016 and December 2017).

The majority of flocks were kept in furnished cages which is likely due to the simpler conversion of conventional cages to this housing system compared to non-cage systems. Fitting into the farm and consumer demands are important factors when choosing new housing systems during the transition period [18,19]. A survey among over 800 consumers in Vancouver, British Columbia, showed a strong increase in consumption of cage-free specialty eggs i.e., 32.9% free range eggs, 11.9% organic eggs, and 7.6% free-run eggs in 2009, compared to a combined consumption of 8% of cage-free specialty eggs in 2007 [20]. This finding shows increased consumer demand for cage-free eggs. While there are some regional differences in consumer preferences and willingness to pay [21,22], it is likely that food trends occurring across Canada such as the use of cage-free eggs in restaurants and more specialized, individual choice in grocery stores will increase and this plays a role in shaping consumer behavior [8].

The recently updated Code of Practice for laying hens in Canada provides interim and final requirements for aspects such as space allowance, nesting, perching, foraging, and dustbathing [11]. Approximately 45% of the flocks in the study were placed in the laying facilities before the Code of Practice came into effect, which could explain why not all practices conformed with requirements set out in the Code at the time of the study. For example, the range of calculated space allowances suggests that all flocks were not yet housed in line with the transitional requirements of 580.6 cm^2^/hen for furnished cages [11]. The space allowances calculated for non-cage housing systems should be considered as approximations; not all useable space (e.g., vertical space in the tiers) was accounted for. Similar to furnished caged flocks, some flocks in non-cage systems did not yet meet the transitional requirements (929.0–1115.0 cm^2^/hen depending on perch space provided). However, average space allowance reveals similar values to the final space allowances required in the Code of Practice [11] and the 750 cm^2^/hen space allowance for furnished cages and stocking density of 9 hens/m^2^ (1111.1 cm^2^/hen) for non-cage systems as set out in the European legislation [1]. It would be interesting to investigate drivers and barriers in uptake and compliance with the new Code of Practice requirements in flocks placed after the transitional date.

Nearly half of the flocks in furnished cages were not provided with a scratch area, possibly due to being installed prior to when the Code of Practice came into effect [11]. These type of cages are generally considered cages with furnishings or enrichable cages and will need to be updated to meet the final requirements of furnished cage housing systems in order to provide a flooring surface for foraging and/or dustbathing [11]. Similarly, over 20% of the non-cage systems did not provide any litter but rather had a fully slatted barn, a practice which is no longer allowed under the Code of Practice [11], in comparison to 3.3% of flocks in Switzerland [23]. In contrast, 34 barn and free range layer farms in Australia all reported to use slatted flooring [6], showing large differences in flooring types in different countries. Additionally, the time at which studies were conducted should be kept in mind, as, for example, the Swiss study was conducted between 1997 and 1998 [23] and should be interpreted with caution as the current conditions in the sector are likely different. In the majority of farms that provided litter, at least one-third of the barn was covered with litter, which is in line with European legislation [1]. While few specific recommendations on the management of litter substrate are available, indicating that litter must be of good quality and friable [11], our findings show most farmers do not break up or replace the litter after first access. Factors such as proper barn climate and functioning drinkers, which minimize water spillage, can help ensure litter stays friable [24,25,26]. Litter depth averaged around 5 cm but ranged from 0.5 to 12.7 cm. According to a survey on laying hen farms in Switzerland, over 80% of flocks were provided with more than 3 cm of litter when 2–3 months after lay, while 21% showed a maximum depth of more than 10 cm [23]. While no figures are available on optimal litter depth, Huber-Eicher and Sebö [27] recommended to use a lower litter depth in general. Instead, they suggested to regularly supplement with new litter to avoid problems with litter getting into the feeder and to keep the foraging substrate attractive to the birds [27]. Additionally, large litter depths can increase the risk of floor eggs which can be managed through adaptations in rearing to allow birds to find and use nest boxes early on [26]. While it is unclear whether farmers added fresh litter or broke up the litter substrate during lay, 94 out 170 laying hen farmers in the UK reported that there was no loose litter at the end of the laying period [24]. This shows the importance of litter management, and by extension barn climate, in order to ensure that litter provision can fulfill its function as a foraging and dustbathing substrate throughout the laying period. Of the farms that provided litter, nearly a quarter listed manure as the substrate. While litter is a defined as a combination of bedding and manure that builds up over time and therefore is allowed under the Code of Practice, it could be argued that only providing manure without additional bedding material should not be considered normal practice [11,25] and is contradictory to consumer expectations and so can influence public perception of litter-based systems [28]. Recently, von Waldburg et al. [29] showed that laying hens prefer to feed and forage in substrates free of manure. However, when no other options for foraging material are available, laying hens spend more time foraging on scratch pads soiled with manure [30], suggesting that manure as a litter substrate is preferred over no litter substrate at all. It is important to consider though the implications the presence of manure in a system can have in terms of egg contamination, foot disorders, and animal or worker health in relation to ammonia [31].

Manure removal from the system occurred at least 1× per week in furnished cage and multi-tier systems, likely due to the ease with which automatic manure belts can be operated. Manure removal occurred less frequently in single-tier systems, especially when the system was a fully slatted barn. These systems often have a manure pit that is only cleared out at the end of the flock. Frequent manure removal can prevent ammonia concentrations from reaching harmful levels, and higher ammonia levels have been recorded in single-tier systems compared to free range and furnished cage systems [32]. Laying hens prefer environments with fresh air over environments with ammonia concentrations of 25 ppm [33]. However, further investigation is required to determine how frequently manure should be removed to avoid these aversive concentrations. Interestingly, Garner et al. [34] found that the number of eggs/hen-housed increased when manure was removed in conventional cage systems during the laying period, suggesting that frequent manure removal can have positive implications not only for worker and animal welfare but also flock productivity.

Little variation was observed in laying hen feeding practices. The majority of flocks were fed mashed feed through chain feeders. Flocks were fed between 2 to 12 times per day (mean: 5.5 times per day), similar to a previous study that reported that the majority of flocks were fed 6–7 times per day [35]. Diet changes were a common occurrence on most farms which was related to the age of the flock, with flocks having undergone more than three diet changes by the time they reached approximately 48 weeks of age. This is largely in line with common breed guidelines [36,37]. Midnight feeding where lights are turned on during the dark period to encourage feed intake can be used to improve egg shell quality [38]. However, turning on the lights during the dark period can also influence sexual maturity, and this practice is generally not recommended for pullets [39]. Additionally, this practice is not allowed in all countries for laying hens. In fact, the European directive 1999/74/CE requires that laying hens have 8 h of uninterrupted darkness every 24 h, which would be disrupted when applying midnight feeding [40]. Similarly, feeding animal by-products was not common, likely due to the potential consequences it can have on food safety and human health [41].

The hours of light per day offered was relatively constant between flocks and the little variation found can be explained by the large age range of the flocks. Light intensity was similar to the recommended 5–15 lux in the breed guidelines [36,37]. However some farmers reported less than 5 lux, and nearly a quarter of farmers could not estimate the lux. A dawn/dusk period was provided in the majority of flocks. Gradual dimming of lights by area was especially used in multi-tier systems, which encourages birds to perch during the night [42].

### 4.2. Flock Characteristics

The majority of breeds included in the study flocks were Dekalb White, Hy-line Brown, ISA Brown, Lohmann Brown, and Lohmann LSL. However not all respondents were able to identify the specific strains. There was an equal distribution of white- and brown-feathered flocks, with white-feathered flocks being more common in furnished cages, while brown-feathered flocks were mostly kept in non-cage systems. Brown-feathered birds lay eggs with brown shell color. Such eggs are perceived by consumers to have a higher nutritional value as well as an association with naturalness, which could explain consumer preference for brown over white eggs [20,43,44]. This perception could further contribute to the use of brown-feathered birds in non-cage housing systems, although breed suitability to the housing system should be considered [45].

All flocks were beak trimmed at day-old, which is a standard practice in order to reduce feather pecking problems [13]. However, beak trimming is under scrutiny and does not reduce feather pecking completely if flock management is not adequate [13]. Another important consideration is the housing system used during rear. In order for flocks to perform successfully during lay, it is recommended to rear pullets in a similar system to that used during lay [45]. This provides birds with opportunities to learn appropriate behaviors (e.g., access to litter, perches, nest boxes) that reduce behavioral problems (e.g., floor eggs, feather pecking) and can improve productivity and reduce mortality [26,46,47]. Most pullet housing systems at the time of the survey were conventional cage systems [15]. Currently there are only few furnished cages for rearing pullets available on the market. In this study, laying hen flocks housed in furnished cages typically originated from conventional cage systems (one flock from furnished cage rearing facility), with the exception of two flocks that were reared in a single-floor system. Janczak and Riber [48] indicated that aviary-reared pullets should not be subsequently housed in furnished cages, however this recommendation was based on small furnished cages housing a maximum of 9 birds. More recent work investigating the effect of aviary-rearing for hens subsequently housed in large 30- or 60-bird furnished cages found few detrimental effects and several benefits to aviary rearing compared to conventional rearing [49]. Nearly 95% of flocks in single-tier (1 flock from conventional cage rearing facility) and 100% of flocks in multi-tier systems were reared in the same system as they were housed in during lay. It is important to note that the majority of flocks in non-cage systems were reared by layer producers, giving them greater control over rearing facilities and possibilities to match conditions between rear and lay, while the majority of flocks in furnished cage systems came from a rearing supplier.

On average birds were inspected 2–3× per day by 1–2 workers on the farm. In comparison, Louton et al. [35] found that 44% of farmers inspected their flock 1 time, 27.2% 2 times and 14.4% 3 times per day. The amount of time spent on inspections in our study was similar to previously reported durations with most farmers spending up to 30 min per inspection [35]. Self-reported practices such as disinfection of barns and use of dedicated clothes and/or boot dips were common. Only a limited number of farmers claimed to have developed and implemented a flock health plan with their veterinarian and veterinarian visits to the farms were generally rare. Poultry farmers in Australia reported similar practices with veterinarian visits occurring once per year or only when a health issue occurred [6]. Higher stocking densities and more frequent bird-to-bird contact in furnished cage and non-cage systems are considered a risk for increased pathogen density and subsequent disease, especially in suboptimal housing and management conditions [45,50]. In spite of this, prevalence of disease in the study flocks was low, likely due to stringent vaccination schemes used on-farm (data not shown). Following the ban on conventional cages in Switzerland, Kaufmann-Bart and Hoop [50] found a decrease in viral diseases over a 12 year period, while bacterial diseases tended to increase. They emphasized the importance of vaccination, farm hygiene, and effective biosecurity in maintaining good health in laying hens in non-cage systems and concluded that health levels were comparable to those observed in caged housing systems [50]. Cumulative flock mortality was below 6% in all study flocks, which is in line with findings by both Louton et al. [35] and by the European Food Safety Authority [45], who found that mortality in the majority of flocks was below 10%. The mortality rate was highest in multi-tier systems, followed by single-tier and furnished cage systems. Birds in furnished cages typically show lower mortality rates compared to those in non-cage systems [32,51]. However, this is dependent on the age and genotype of the birds, management, and disease risk [45].

Flock performance and egg productivity are dependent on the flock age. In this cross-sectional study, flocks were observed at different stages of lay making comparison between flocks difficult. However, 50% rate of lay and peak production observed in these different flocks were comparable to those stated in the breed guidelines [36,37]. Birds in non-cage systems showed a higher feed consumption and lower productivity compared to furnished caged birds as reported in previous studies [45,47,52]. This was, however, confounded by strain since the majority of furnished caged flocks were white-feathered, whereas those in non-cage systems were brown-feathered birds.

## 5. Conclusions

This study is the first to describe current housing design and management practices used on Canadian farms. In particular, furnished cage or non-cage housing systems were surveyed. By better understanding current and developing management practices, the transition away from conventional cage systems can be facilitated. The importance of litter management, including the maintenance of litter quality, and development of a flock health plan, including vaccination, hygiene, and biosecurity practices, are areas of potential improvement. Further research is needed to make clear recommendations in these areas.

## Figures and Tables

**Table 1:** A description of general farm characteristics for 65 laying hen flocks placed between October 2016 and December 2017 according to housing system (furnished cage n = 26, single-tier n = 17, multi-tier n = 22) in Canada.

Variable	Furnished Cage		Single-Tier		Multi-Tier	
	n	%	n	%	n	%
No. of hens						
Less than 1000	1	3.9	1	5.9	0	0.0
1000–5000	1	3.9	2	11.8	0	0.0
5000–10,000	4	15.4	6	35.3	0	0.0
10,000–15,000	9	34.6	2	11.8	3	13.6
15,000–20,000	2	7.7	0	0.0	1	4.6
20,000–25,000	5	19.2	2	11.8	4	18.2
More than 25,000	4	15.4	4	23.5	14	63.6
Age of system						
Less than 1 years old		15.4	5	33.3	5	22.7
1–4 years old	11	42.3	5	33.3	10	45.5
5–10 years old	9	24.6	2	13.3	6	27.3
More than 10 years old	2	7.7	3	20.0	1	4.6

**Table 2:** Distribution of perches and enrichment provision for 65 laying hen flocks placed between October 2016 and December 2017 according to housing system (furnished cage n = 26, single-tier n = 17, multi-tier n = 22) in Canada.

Variable	Furnished Cage		Single-Tier		Multi-Tier	
	n	%	n	%	n	%
Perches						
Yes	25	96.2	12	75.0	22	100.0
No	1	3.9	4	25.0	0	0.0
Perches at different heights ^1^						
Yes	5	20.0	11	91.7	22	100.0
No	20	80.0	1	8.3	0	0.0
Space to perch at the same time ^1^						
Yes	19	82.6	8	72.7	18	85.7
No	4	17.4	3	27.3	3	14.3
Enrichment provided						
Yes	0	0.0	6	37.5	8	36.4
No	25	100.0	10	62.5	14	63.6

^1^ Only answered by farmers who indicated that they provided perches.

**Table 3:** Summary of litter management aspects for laying hen flocks placed between October 2016 and December 2017 according to housing system (single-tier n = 17, multi-tier n = 22) in Canada.

Variable	Single-Tier		Multi-Tier	
	n	%	n	%
Type of barn				
All litter barn	2	12.5	6	28.6
Combination of wire, slats and litter	6	37.5	15	71.4
All wire/slatted barn	8	50.0	0	0.0
Proportion of barn with litter ^1^				
<1/3 of barn	2	50.0	1	6.7
1/3 of barn	1	25.0	5	33.3
>1/3 of barn	1	25.0	9	60.0
Type of litter ^2^				
Sawdust	4	44.4	2	10.5
Sand	0	0.0	1	5.3
Wood shavings	2	22.2	10	52.6
Straw	1	11.1	0	0.0
Combination of the above	1	11.1	0	0.0
None/manure	1	11.1	6	31.6
Litter replaced after first access ^2^				
Yes	2	25.0	7	33.3
No	6	75.0	14	66.7
Frequency of breaking up of litter ^2^				
Never	7	87.5	11	57.9
Seasonally	0	0.0	1	5.3
Monthly	1	12.5	0	0.0
Weekly	0	0.0	4	21.1
Daily	0	0.0	3	15.8

^1^ Only answered by farmers who indicated that they had a barn with ‘combination of wire, slats and litter’. ^2^ Excluded farmers who indicated that they had an ‘all wire/slatted barn’.

**Table 4:** Frequency of nutrition and feeding practices for 65 laying hen flocks placed between October 2016 and December 2017 according to housing system (furnished cage n = 26, single-tier n = 17, multi-tier n = 22) in Canada.

Variable	Furnished Cage		Single-Tier		Multi-Tier	
	n	%	n	%	n	%
Feed structure						
Mashed feed	18	69.2	17	100.0	15	68.2
Pelleted feed	3	11.5	0	0.0	2	9.1
Grains	0	0.0	0	0.0	0	0.0
Crumbs	5	19.2	0	0.0	5	22.7
Feed source						
Home-milled	11	42.3	5	29.4	9	40.9
Purchased	15	57.7	12	70.6	13	59.1
No. of times diet has been changed						
No changes	3	12.0	4	23.5	5	23.8
1×	4	16.0	1	5.9	3	14.3
2×	2	8.0	1	5.9	2	9.5
3×	5	20.0	7	41.2	4	19.1
4×	7	28.0	3	17.7	3	14.3
More than 4×	4	16.0	1	5.9	4	19.1
Method of changing diet ^1^						
Gradual change	19	82.6	12	92.3	12	70.6
Immediate change	4	17.4	1	7.7	5	29.4
Midnight feeding						
Yes	4	15.4	1	6.3	0	0.0
No	22	84.6	15	93.8	22	100.0
Supplements provided						
No	7	29.2	6	37.5	4	19.1
Yes	17	70.8	10	62.5	17	81.0
Insoluble grit ^2^	4	16.7	2	12.5	5	23.8
Insoluble fibre ^2^	6	25.0	5	31.3	8	38.1
Oyster shell ^2^	8	33.3	6	37.5	6	28.6
Vitamins ^2^	13	54.2	8	50.0	14	66.7
Animal by-products in feed						
Yes	4	19.1	2	13.3	4	21.1
No	17	81.0	13	86.7	15	79.0

^1^ Only answered by farmers who indicated that they had changed the diet of the flock. ^2^ Those farmers who indicated that provided supplements were asked to indicate if they provided: insoluble grit, insoluble fiber, oyster shell, and vitamins.

**Table 5:** Frequency of environmental control practices for 65 laying hen flocks placed between October 2016 and December 2017 according to housing system (furnished cage n = 26, single-tier n = 17, multi-tier n = 22) in Canada.

Variable	Furnished Cage		Single-Tier		Multi-Tier	
	n	%	n	%	n	%
Type of lighting						
Incandescent	4	15.4	5	29.4	1	5.0
Fluorescent	3	11.5	0	0.0	0	0.0
LED	19	73.1	12	70.6	19	95.0
Dawn/dusk period provided						
Yes	24	92.3	10	58.8	20	90.9
No	2	7.7	7	41.2	2	9.1
Method of dawn/dusk provision ^1^						
All lights dimmed	20	83.3	9	90.0	6	30.0
Gradual dimming by area	4	16.7	1	10.0	14	70.0
Light intensity						
Less than 5 lux	2	10.0	1	7.1	2	13.3
5–10 lux	9	45.0	7	50.0	3	20.0
11–15 lux	2	10.0	2	14.3	7	46.7
16–20 lux	3	15.0	2	14.3	2	13.3
21–25 lux	3	15.0	2	14.3	1	6.7
More than 25 lux	1	5.0	0	0.0	0	0.0
Type of ventilation						
Controlled (fan)	15	96.2	15	88.2	19	86.4
All natural	0	0.0	0	0.0	1	4.6
Combination (fan + natural)	1	3.9	2	11.8	2	9.1
Manure removal						
1× per day	1	4.0	1	6.7	3	13.6
3× per week	2	8.0	1	6.7	3	13.6
2× per week	12	48.0	1	6.7	13	59.1
1× per week	10	40.0	0	0.0	3	13.6
1× or more per quarter	0	0.0	2	13.3	0	0.0
End of flock	0	0.0	10	66.7	0	0.0

^1^ Only answered by farmers who indicated that they provided a dawn/dusk period.

**Table 6:** Frequency of flock characteristics for 65 laying hen flocks placed between October 2016 and December 2017 according to housing system (furnished cage n = 26, single-tier n = 17, multi-tier n = 22) in Canada.

Variable	Furnished Cage		Single-Tier		Multi-Tier	
	n	%	n	%	n	%
Feather colour						
Brown	6	23.1	12	75.0	14	63.6
White	20	76.9	4	25.0	8	36.4
Breed						
Bovans White	1	4.4	0	0.0	0	0.0
Dekalb White	8	34.8	1	8.3	1	5.6
Hy-line Brown	1	4.4	4	33.3	1	5.6
ISA Brown	3	13.0	1	8.3	2	11.1
Lohmann Brown Classic	0	0.0	1	8.3	1	5.6
Lohmann Brown Lite	1	4.4	2	16.7	8	44.4
Lohmann LSL Classic	1	4.4	0	0.0	1	5.6
Lohmann LSL Lite	8	34.78	2	16.7	3	16.7
Novogen	0	0.0	1	8.3	1	5.6

**Table 7:** Description of flock rearing and placement practices for 65 laying hen flocks placed between October 2016 and December 2017 according to housing system (furnished cage n = 26, single-tier n = 17, multi-tier n = 22) in Canada.

Variable	Furnished Cage		Single-Tier		Multi-Tier	
	n	%	n	%	n	%
Source						
Home-reared	7	26.9	9	52.9	15	68.2
Rearing supplier	15	57.7	8	47.1	7	31.8
Combination	4	15.4	0	0.0	0	0.0
Visit rearing flock						
Yes	9	36.0	11	68.8	13	61.9
No	16	64.0	5	31.3	8	38.1
Birds all from the same rearing flock						
Yes	21	80.8	15	88.2	22	100.0
No	5	19.2	2	11.8	0	0.0
Housing system during rear						
Conventional cage	20	87.0	1	6.3	0	0.0
Enriched cage	1	4.3	0	0.0	0	0.0
Single tier	2	8.7	15	93.8	0	0.0
Multi tier	0	0.0	0	0.0	22	100.0
Match conditions barn to rearing						
No	10	61.5	2	11.8	0	0.0
Yes	16	38.5	15	88.2	21	100.0
Housing ^1^	3	18.8	13	86.7	18	85.7
Perches ^1^	0	0.0	8	53.3	15	71.4
Litter ^1^	0	0.0	5	33.3	15	71.4
Feed ^1^	14	87.5	14	93.3	19	90.5
Environment ^1^	14	87.5	10	71.4	15	71.4

^1^ Those farmers who indicated that they matched conditions between rearing and laying facilities were asked whether they do this for the following aspects: housing, perches, litter, feed and environment.

**Table 8:** Frequency of flock health management practices for 65 laying hen flocks placed between October 2016 and December 2017 according to housing system (furnished cage n = 26, single-tier n = 17, multi-tier n = 22) in Canada.

Variable	Furnished Cage		Single-Tier		Multi-Tier	
	n	%	n	%	n	%
No. of bird inspections per day						
1×	4	15.4	0	0.0	1	4.6
2×	13	50.0	10	58.8	8	36.4
3×	2	7.7	6	35.3	8	36.4
4×	3	11.5	1	5.9	2	9.1
5×	0	0.0	0	0.0	1	4.6
More than 5×	4	15.4	0	0.0	2	9.1
No. of workers						
1 worker	11	42.3	3	17.7	11	50.0
2 workers	12	46.2	14	82.4	11	50.0
3 workers	3	11.5	0	0.0	0	0.0
More than 3 workers	0	0.0	0	0.0	0	0.0
Average time spent on inspection per day						
Less than 15 min	1	3.9	0	0.0	0	0.0
15–30 min	4	15.4	8	50.0	4	19.1
30–45 min	8	30.8	4	25.0	1	4.8
45–60 min	8	30.8	1	6.3	10	47.6
More than 60 min	5	19.2	3	18.8	6	28.6
Vary route during inspections						
Yes	15	57.7	7	43.8	13	65.0
No	11	42.3	9	56.3	7	35.0
Flock health plan implemented						
Yes	6	23.1	5	35.7	6	31.6
No	20	76.9	9	64.3	13	68.4
Diseases observed since placement						
Yes	4	16.0	3	18.8	4	20.0
No	21	84.0	13	81.3	16	80.0

**Table 9:** Production parameters for 65 laying hen flocks placed between October 2016 and December 2017 according to housing system (furnished cage n = 26, single-tier n = 17, multi-tier n = 22) in Canada.

Variable	Furnished Cage				Single-Tier				Multi-Tier			
	n	Mean	Stddev	Range	n	Mean	Stddev	Range	n	Mean	Stddev	Range
Flock age (wks)	26	43.6	15.77	21–69	17	44.4	13.49	20–68	22	47.4	14.33	19–67
Egg production rate (%)	25	90.5	11.16	55–99	17	86.9	20.43	10–98	20	89.4	15.94	23–98.5
Peak production rate (%) ^1^	22	97.6	1.25	95–99.2	12	95.5	2.18	92–99	19	97.0	1.57	94–100
Age at 50% rate of lay (wks) ^1^	22	20.8	0.81	19–22	13	21.4	1.18	20–24	18	20.6	0.90	18–22
Age at peak production (wks) ^1^	20	26.3	3.27	22–35	13	26.3	4.11	23–36	19	27.2	3.61	23–35
Feed consumption (gr/day/bird)	25	106.8	6.46	90–119	14	110.5	6.58	99–120	21	112.0	7.10	98–125.1

^1^ Only answered when birds had reached 50% rate of lay or peak production.
